# Medication Adherence and Perceptions According to the Presence or Absence of a Migration Background in a Dialysis Unit (DIANA Qualitative Study)

**DOI:** 10.2147/PPA.S503025

**Published:** 2025-07-18

**Authors:** Jennifer Dotta-Celio, Mélanie Lelubre, Sabrina Bolzon, Georges Halabi, Michel Burnier, Patrick Bodenmann, Menno Pruijm, Marie P Schneider

**Affiliations:** 1 Department of Ambulatory Care, Pharmacy, Unisanté, Center for Primary Care and Public Health & University of Lausanne, Lausanne, Switzerland; 2 School of Pharmaceutical Sciences, University of Geneva, Geneva, Switzerland; 3 Institute of Pharmaceutical Sciences of Western Switzerland, University of Geneva, University of Lausanne, Geneva, Switzerland; 4 Service of Nephrology and Hypertension, Department of Medicine, Lausanne University Hospital and University of Lausanne, Lausanne, Switzerland; 5 Faculty of Biology and Medecine, University of Lausanne, Lausanne, Switzerland; 6 Department of Vulnerabilities and Social Medicine, Unisanté, Center for Primary Care and Public Health & University of Lausanne, Lausanne, Switzerland; 7 Chair of medicine for Vulnerable Populations, Faculty of Biology and Medicine, University of Lausanne, Lausanne, Switzerland

**Keywords:** patient adherence, renal dialysis, polypharmacy, culture, migrants

## Abstract

**Purpose:**

Migration status has received little attention in the context of medication adherence. This qualitative study aims to consolidate the findings of the quantitative DIANA study by: 1) exploring medication management, medication knowledge and perceptions and 2) identifying whether there are differences in medication management, knowledge and perceptions between patients according to the patients’ migration status.

**Patients and Methods:**

This monocentric qualitative study was part of the research protocol DIANA (*Dialyse chronique et adhésion thérapeutique*). Patients were recruited at the chronic dialysis unit of the Lausanne University Hospital. In-depth, face-to-face interviews were conducted; for allophone patients, an interpreter was present. Each interview was recorded and transcribed verbatim. A content analysis was performed.

**Results:**

Eighteen interviews were performed. Nine patients were Swiss, 3 had a residence/settlement permit, 6 were provisionally admitted/asylum seekers. Six interviews were performed with an interpreter. Core themes were: (1) treatment management (medication intake had become a habit, forgetting was rare), (2) heterogeneous treatment knowledge but highly perceived necessity, (3) reported medication disadvantages were pill burden and side effects, (4) role of the patient in the medical environment (trust in physicians but patients making decisions regarding their treatment on their own, without consulting healthcare professionals). Swiss patients engaged more in shared decision-making and were more likely to talk about side effects than migrant patients. Provisionally admitted foreign nationals/asylum seekers talked more about their gratitude to the healthcare system.

**Conclusion:**

More studies are needed to understand how shared decision-making can be established between health professionals and patients, taking into account their migration status. To provide tailored education, it is essential to evaluate patients’ health literacy and to use the teach-back method to find the best way to convey health messages. The acquisition of transcultural clinical skills by health professionals is necessary to support medication adherence.

## Video Abstract

**Figure:**
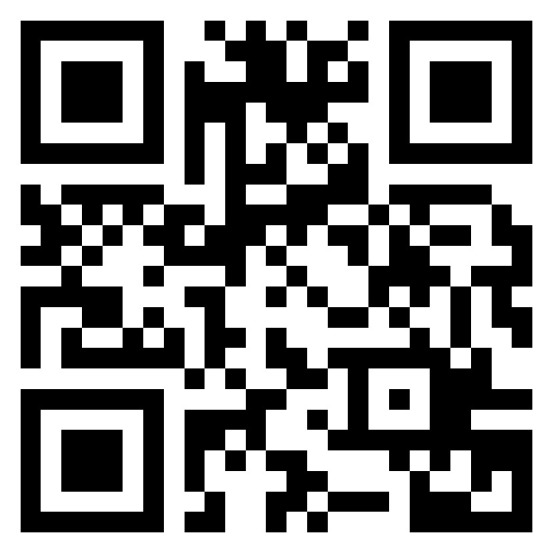

Point your SmartPhone at the code above. If you have a QR code reader the video abstract will appear. Or use:

https://youtu.be/An3DjVzjMCs

## Introduction

According to the European Renal Association (ERA) report, in 2021, the incidence of kidney replacement therapy was 145 per million population; 56% of patients received haemodialysis, 5% received peritoneal dialysis, and 39% were living with a functioning graft.^1^ In Switzerland approximately 5’000 patients were dialyzed in 2018 (no updated data since).^2^ Four elements are fundamental for patients to achieve good health outcomes: medication adherence, participation in regular dialysis sessions, fluid restriction and diet.^3–9^ Medication adherence is the process by which patients take their medications as prescribed.^10^ It is characterized by three constructs: initiation, implementation and persistence.^10^ The first dose taken delineates the initiation. Implementation describes the patient’s daily taking (eg dosage, regimen, timing and other specificities). Persistence corresponds to the period between initiation and discontinuation; discontinuation coincides with a definitive stop of the medication intake.^10^

Medication nonadherence ranges from 3–12.5% to 80–98.6% in patients on hemodialysis (HD),^11^,^12^ while it ranges from 3.9% to 43.0% in patients on peritoneal dialysis (PD).^11^,^13^ Medication nonadherence is associated with a higher risk of mortality and hospitalization in patients.^13^,^14^ Patient beliefs play a key role in adherence. Beliefs of patients about their illness and/or their medications have been measured by means of questionnaires,^12^,^15–18^ but few studies have looked in depth at patients’ perceptions of their medications.^19–23^ Psychosocial factors such as immigrant status, poverty, perceived identity loss and family dysfunction were associated with treatment nonadherence in Mexican-American women on hemodialysis in Texas.^24^ Misinformation, limited education and issues with maintenance were identified as barriers to home dialysis among Latin patients with kidney disease.^25^ In Switzerland, several studies have examined the health of forced and international migrants,^26–30^ but no study evaluated the medication adherence of migrants on long-term dialysis.

In Switzerland, at the end of 2015 (year of the DIANA study), 24.9% of the people living in the country were non-nationals.^31^ In the 1990s, several tens of thousands of Kosovars fled the war in the former Yugoslavia, seeking political asylum, particularly in Switzerland.^32^ Obtaining Swiss nationality is not immediate; about one-fifth of the foreign population is born in Switzerland but still holds their original nationality.^33^ At the end of 2022, Switzerland had a total of 1’415’231 holders of settlement permits (C permits) and 809’974 holders of residence permits (B permits).^34^ In 2025, 16’271 asylum seekers (N permits) and 42’545 provisionally admitted foreigners (F permits) were present in Switzerland.^35^

The aim of the DIANA study was therefore to assess the quantitative and qualitative aspects of medication adherence in patients on dialysis according to their migration status. The quantitative study, which will be published separately, revealed differences in medication persistence; notably, the migrant population had higher persistence.^36^ Beliefs about medicines were not significantly different between Swiss+Resident/Settled foreign nationals and migrants.^36^ The qualitative study aims to consolidate the findings by: 1) exploring medication management, patients’ knowledge and perceptions of medication and 2) identifying whether there are differences in medication management, knowledge and perceptions between patients according to their migration status.

## Materials and Methods

This article was written according to COREQ^37^ and EMERGE^38^ guidelines. Written informed consent was obtained prior to inclusion. Patients were informed and agreed that the interview was recorded and transcribed. They were also informed that all data were coded before being analyzed. The DIANA study was authorized by the Local Ethics Commission (Canton Vaud, Switzerland, Study N. 35/15). All procedures performed involving human participants were in accordance with the 1964 Helsinki declaration and its later amendments.

### Research Team and Reflexivity

The interviews were all conducted by JDC, a pharmacist trained in qualitative research. Prior to the study, JDC collaborated with the chronic dialysis unit as a pharmacist; some patients had clinical interactions with her before this study. Patients were informed that the researchers were interested in their medication management, knowledge and perceptions. The other coders (ML and MPS) were experienced in qualitative analysis. SB coded under the supervision of JDC. MPS and ML did not have any clinical role and did not meet with the patients. SB collected the quantitative data.^36^

### Theoretical Frameworks

The interview guide categories (see Appendix 1) were developed based on the World Health Organization (WHO) model that classified medication adherence determinants in five dimensions (patient-, therapy-, condition-, socio-economic-, healthcare team- and system-related factors),^39^ and on the social-cognitive theory,^40^,^41^ particularly the Information-Motivation-Behavioral skills model (IMB Model).^39^

### Setting and Participant Selection

Patients were recruited at the chronic dialysis unit of the Lausanne University Hospital, during a dialysis session or after a medical visit (patients on peritoneal dialysis). Note that in Switzerland, health insurance is mandatory and medications are reimbursed according to a national list. Patients who were dialyzed since at least three months in the chronic dialysis unit were all invited to participate to the DIANA study. The three months cut off was used to assure that only chronic dialysis patients were included. This period was also necessary to be able to assess adherence through medication refills for the quantitative part of the study. Patients who participated in the DIANA quantitative study^36^ were asked to participate hereafter to this qualitative sub-study. Patients were divided in three groups: a) Swiss nationality (Group-1), b) resident foreign nationals (C permit) and settled foreign nationals (B permit) (Group-2), c) provisionally admitted foreign nationals (F permit) and asylum seekers (N permit) (Group-3) (see Table 1).

**Table 1:** Patient Classification According to the Migrant Status in Switzerland

Classification	Permit	Definition
Swiss patient	Swiss passport	Patients with the Swiss nationality
Resident foreign nationals	Permit B	The residence permit is valid for five years and is renewable. It is issued if the foreign national is in possession of an employment contract for at least twelve months or for unlimited duration
Settled foreign nationals	Permit C	A settlement permit is granted after five or ten years’ residence in Switzerland. The right to settle in Switzerland is not subject to any time restrictions or conditions
Provisionally admitted foreigners	Permit F	Persons who have been ordered to quit Switzerland and return to their native countries but in whose cases the enforcement of this order has proved inadmissible (violation of international laws), unreasonable (endangerment) or impossible (for technical reasons). This provisional admission constitutes a substitute measure for a duration of twelve months, extendable for another twelve months at a time
Asylum-seekers	Permit N	Persons who have applied for asylum in Switzerland and whose application is being processed

Patients were interviewed during dialysis sessions or in a confidential room at the community pharmacy of the Center for Primary Care and Public Health, according to their preference. When the interviews were carried out during dialysis sessions, other persons were present in the room: other patients (four-person room), a nurse and sometimes medical doctors. Closed curtains around each patient’s bed guaranteed a certain level of confidentiality, and the investigator made sure that each interviewee felt comfortable.

Maximal variation sampling was used, ensuring adequate representation of variety of patients of the chronic dialysis unit with respect to gender, nationality, mother tongue, age, time spent in dialysis, and being on the transplant list or not. In case patients did not speak French, a community interpreter presented the study to them and was present during the interview for translation.

### Data Collection

A semi-structured interview guide (see Appendix 1) was developed and used to guide the individual, in depth, semi-structured interview. The categories were: (a) treatment knowledge, (b) treatment management, (c) facilitators and barriers to treatment, (d) need for information, (e) social support for treatment, (f) forgetting doses, (g) motivation to take treatment, and (h) treatment perceptions. Topics (e), (f), (g) and (h) were explored only if mentioned by patients. The individual interviews took place once and were not repeated.

Field notes were immediately transcribed after each interview to describe the setting, the number and the role of the persons present in the room. Interviews were audiotaped and transcribed verbatim. The transcripts were not returned to patients for validation.

### Data Analysis

A content analysis was performed deductively. Two coders (JDC and ML, or JDC and MPS, or JDC and SB) worked independently on each transcript and identified themes. In the beginning of the coding process, a consensus meeting was organized to build a preliminary codebook. This codebook was used to code transcripts of subsequent interviews and was updated when new codes emerged. The final codebook was then used to recode all transcripts before the process was considered as complete. The analysis was performed in Word^®^ and Excel^®^. Particular attention was paid to divergent cases, to better understand the participants’ diverse perceptions.

For the “knowledge” category, the patients’ quotes were compared to the data collected in the medical chart (ie each prescribed medication and its therapeutic indication). This process allowed us to determine whether patients’ knowledge was fully (the patient knew all the prescribed medication names and all the therapeutic indications), partially (the patient knew some names and indications) or insufficiently aligned with medical information (triangulation).^42^

## Results

Forty-five out of 76 eligible patients (59%) accepted to participate to the DIANA quantitative study,^36^ and 33 of them accepted to participate to this qualitative sub-study. Eighteen interviews were performed with 16 patients undergoing hemodialysis and two patients undergoing peritoneal dialysis. These 18 patients represented the diversity of the dialysis population according to the predefined categories; as data saturation had been reached after 18 interviews, no further ones were performed. Interviews were performed between March and September 2015, either during a dialysis session (n = 16) or in an office located in the community pharmacy of the primary care center (n = 2). The median interview duration was 40 minutes, with a range from 23 to 60 minutes. Six interviews took place in the presence of a community interpreter. All the patients’ characteristics are presented in Tables 2–4.

**Table 2:** Patients’ Socio-Demographic Characteristics

	All	Swiss Nationality	Resident Foreign Nationals and Settled Foreign Nationals (B/C Permit)	Provisionally Admitted Foreign Nationals and Asylum Seekers (F/N Permit)
Patients	n = 18	n_1_= 9	n_2_= 3	n_3_ = 6
Sex				
*Male sex*, n *(%)*	12 (67%)	7 (78%)	2 (67%)	3 (50%)
Age (years), median [1^st^-3^rd^ quartiles]	56.5 [48–65]	65 [53–72]	62 [53–64]	46 [42–28]
Years in Switzerland, median [1^st^-3^rd^ quartiles]	38 [6–65]	65 [49–72]	38 [14–40]	5.5 [4–6]
Employment				
*Employed*, n *(%)*	4 (n_obs_= 15) (27%)	2 (n_obs_= 8) (25%)	2 (n_obs_= 3) (66%)	0 (n_obs_= 4) (0%)

**Table 3:** Patients’ Clinical Characteristics

	All	Swiss Nationality	Resident Foreign Nationals and Settled Foreign Nationals (B/C Permit)	Provisionally Admitted Foreign Nationals and Asylum Seekers (F/N Permit)

Patients	n = 18	n_1_= 9	n_2_= 3	n_3_= 6
Dialysis				
*Hemodialysis*, n *(%)*	16 (89%)	7 (78%)	3 (100%)	6 (100%)
*Peritoneal dialysis*, n *(%)*	2 (11%)	2 (22%)	0 (0%)	0 (0%)
Time spent on dialysis (months), median [1^st^-3^rd^ quartiles]	38 [22–100]	34 [17–40]	22 [16–62]	71.5 [37–102]
Transplant list				
*Yes*, n *(%)*	11 (61%)	6 (67%)	1 (33%)	4 (67%)

**Table 4:** Patients’ Medication Characteristics

	All	Swiss Nationality	Resident Foreign Nationals and Settled Foreign Nationals (B/C Permit)	Provisionally Admitted Foreign Nationals and Asylum Seekers (F/N Permit)

Patients	n = 18	n_1_= 9	n_2_= 3	n_3_= 6
Nr of oral medications per patient (nr of active substances), median [1^st^-3^rd^ quartiles]	11 [8–13]	11 [7–13]	8 [7–12]	11 [10–12]
*Phosphate and potassium binders* n_medications_ *= 3* n_medications_ *= 2* n_medications_ *= 1*	5 (28%) 5 (28%) 3 (17%)	3 (33%) 2 (22%) 1 (11%)	1 (33%) 0 (0%) 1 (33%)	1 (17%) 3 (50%) 1 (17%)
*Analgesics* n_medications_ *= 2* n_medications_ *= 1*	3 (17%) 9 (50%)	1 (11%) 2 (22%)	0 (0%) 3 (100%)	2 (33%) 4 (67%)
*Antihypertensives* *Alpha- and beta-blocker* n_medications_ *= 1* *Angiotensin II receptor antagonists* n_medications_ *= 1* *Beta-blocker* n_medications_ *= 1* *Angiotensin converting enzyme inhibitors* n_medications_ *= 1*	3 (17%) 5 (28%) 9 (50%) 3 (17%)	3 (33%) 1 (11%) 2 (22%) 2 (22%)	0 (0%) 1 (33%) 2 (67%) 0 (0%)	0 (0%) 3 (50%) 5 (83%) 1 (17%)
*Anticoagulants, antiaggregants, thrombolytics* n_medications_ *= 1*	11 (61%)	7 (78%)	1 (33%)	3 (50%)
*Proton-pump inhibitors* n_medications_ *= 1*	12 (67%)	4 (44%)	2 (67%)	6 (100%)

### Themes

The most cited themes were: (1) treatment management, (2) treatment knowledge and perceived treatment necessity, (3) disadvantages of medications, (4) patient and medical environments. Some themes and subthemes (forgetting to take medication, patient and medical environments, migration background) were not present initially in the interview guide but the patients spontaneously brought them up.

### Treatment Management

The theme, subthemes and the illustrative quotes are presented in Table 5.

**Table 5:** Theme 1 Treatment Management, and Subthemes with Illustrative Quotes

Themes and Subthemes	Illustrative Quotes
Routine	*D1*:“[Taking medications] when I wake up in the morning has become a habit. I wake up in the morning, I eat breakfast and I immediately take my medications. […] Like when I was a smoker, I finished eating and I smoked a cigarette”. *(Man, North Africa, Swiss nationality, more than 10 years in dialysis)* *D2*: “At first I have to measure the blood sugar […] And then I do the injection for the sugar and after that I have to eat and after I take the medication”. *(Woman, Southeast Europe, F/N permit, 6–10 years in dialysis)*
Storage and organization systems	*D3*: “The box is on a piece of furniture, so I see them [the medications] all the time in front of me. I do not forget them. Before they were in the pharmacy box in the bathroom […] but now I put them on a piece of furniture in the living room […] so I see them all the time […] they are all open access, easy access”. *(Man, Southern Europe, B/C permit,0–5 years in dialysis)* *D4*: “Because I am hyper-organized with my weekly box […] Every Sunday, I fill in all my little boxes […] and then every morning I take the box of the corresponding day, which I put […] where I eat […] Every Sunday morning, it is my little mission”. *(Woman, Swiss, 0–5 years in dialysis)* *D5*: “I like the weekly box, it suits me […] at least I see when I don’t take the medication, I see immediately […] these are the positive aspects, there are also negative ones […] no choice, I don’t feel free […] I feel a little disabled”. *(Man, Southern Europe, B/C permit, 0–5 years in dialysis)*
Forgetting (mentioned spontaneously by patients)	*D6*: “I usually make mistakes when my days are completely disorganized. […] When they ask me to come to the hospital at 9 a.m. this implies that I have to get up at 7 a.m. which I’m not used to. […] [Forgetting] is never voluntary”. *(Man, Swiss, 0–5 years in dialysis)* *D5*: “I sometimes do forget [involuntary], other times I don’t wish [to take my medication] […] sometimes I get tired of taking all these medications […]”. *(Man, Southern Europe, B/C permit, 0–5 years in dialysis)*

#### Routine

Half of the patients said spontaneously that medication intake had become a habit and/or were able to detail the usual sequencing of their daily medication management. The medication intake was associated with meals in half of the patients. Few patients used metaphors to better describe this daily medication management: taking the medications was compared to “eating”, “smoking a cigarette”, “washing your teeth” or “putting the belt on”.

#### Storage and Organization Systems

Interviewees stored their treatment in different places and described various organization systems. The medications were stored either in the living room, in the kitchen, in the bathroom, or in the bedroom. Their precise location was, for example, on the table, on the night table, on the desk, on a piece of furniture, near the window or in small drawers dedicated to the medications. Many patients had freely chosen a weekly box to organize their medication. A patient talked about the positive aspects associated with the weekly box but also about the negative perceived meaning associated to its use, which he had to overcome (eg “I don’t feel free […], I feel a little disabled”).

#### Forgetting

For half of the patients, forgetting was perceived as rare and occurred only in special circumstances (particularly in case of disorganization or when patients were out of home). Few patients said that they never forgot their medication and one patient made the distinction between voluntary missed doses (because of pill fatigue, medication burden) and involuntary mistakes (forgotten doses).

### Knowledge and Perceived Medication Necessity

The theme, subthemes and the illustrative quotes are presented in Table 6.

**Table 6:** Theme 2: Knowledge and Perceived Medication Necessity, and Subthemes with Illustrative Quotes

Themes and Subthemes	Illustrative Quotes
Knowledge of the names of the medications and their indications	*D1 (high level of knowledge)*: “I take carvedilol^a^ it is for my blood pressure. I take acetylsalicylic acid^a^. I take sevelamer^a^. I also take a medication for […] cholesterol […] Statins. I think. Atorvastatin* or something like that”. *(Man, North Africa, Swiss nationality, more than 10 years in dialysis)* *D7 (intermediate level of knowledge with only a few medications mentioned)*: “I have medications to control the phosphate […] and potassium […] I have sodium bicarbonate”. *(Man, Swiss, 0–5 years in dialysis)* *D5 (low level of knowledge)*: “I do not remember the name now but I know […] and I take a yellow stuff that I do not know for what it is […] There are surely others but I do not’ remember […] I am not very sure what it is used for […] they have already explained me but there are so many tablets and I forgot”. *(Man, Southern Europe, B/C permit, 0–5 years in dialysis)*
Perceived medication necessity	*D8*: “I should not forget them, because otherwise it damages the graft so I prefer to take them [medications] correctly […] it makes life possible”. *(Man, Swiss, 0–5 years in dialysis)* *D9*: “Where would I be without these medications […] I mean, every day there is a time when I have to take my medications and this is the sine qua non condition to get better, it’s very clear”. *(Male, Western Asia, F/N permit, 0–5 years in dialysis)*

**Note**: *^a^*The patient mentioned the brand names of the medications, which have been replaced by the International Nonproprietary Names (INN).

#### Patient Knowledge About Their Medications

Levels of patients’ knowledge were variable. Some patients knew all the names and indications of their medications. Others had a partial knowledge as they mentioned only some medications. Some patients had difficulties recalling their names and indications.

#### Perceived Medication Necessity

Many patients were aware of the importance of taking their treatment correctly. For few patients, this issue represented the tension between life and death, for example, they recognized the dramatic impact of medication nonadherence on the function of vital organs such as the heart or lungs. Interestingly, during the interview, a few patients spontaneously projected themselves into a virtual situation of nonadherence in order to explicit their perceived risks of such a hypothetical behavior. A few patients ranked the medications in order of importance (the immunosuppressive medications and the antihypertensive medications were classified as the most important) but some other emphasized that all medications were important.

### Disadvantages of Medication

The theme, subthemes and the illustrative quotes are presented in Table 7.

**Table 7:** Theme 3: Disadvantages of Medications, and Subthemes with Illustrative Quotes

Themes and Subthemes	Illustrative Quotes
Pill burden	*D10*: “The first time there was a lot of medications, there were too many, I thought – my God what they found for me – […] I was scared, it was very difficult […] but now that the treatment is getting better, I feel good […] I am in a good mood, my health condition is good […] If I do not take them [the medications] regularly and correctly, I might not have all these opportunities”. *(Woman, East Africa, F/N permit, 0–5 years in dialysis)*
Side effects	*D1*: “I see that with time the stomach for example starts to become a little sensitive with that [the medication]. And I’m more stressed because of the medication”. *(Man, North Africa, Swiss nationality, more than 10 years in dialysis)* *D11*: “The potassium causes constipation, after that it poses other problems, so it is better to be slightly above the norm [clinical value] when it is not very, very, very serious, if not, I have problems [side effects] every time” *(Man, Swiss, more than 10 years in dialysis)*
Concerns about medications but trust in the physicians	*D12*: “I don’t know, I sometimes think […] there is a contraindication […] I don’t know, I don’t know but I think the doctor he does, he knows better […] if there is a problem he can tell me” *(Man, Southeast Europe, F/N permit, 0–5 years in dialysis)* *D4*: “All these drugs that I take, it’s possible that one day they will generate something else […] a kind of poisoning […] but I want to believe that physicians know exactly what I am taking, and they know if it’s exaggerated or not, or if it’s better to stop […] I trust them […] because if we do not trust them, well it is useless to visit a physician^”^. *(Woman, Swiss, 0–5 years in dialysis)*

The most quoted disadvantages of medication were pill burden and side effects. Half of the patients spoke about pill burden. Another half of the patients described side effects associated with their medications. Patients talked about present or past unpleasant symptoms, which affected negatively their daily life (eg they increased patient’s level of stress). They also feared potential future side effects (eg “a kind of poisoning”).

### Patient and Medical Environments

The theme, subthemes and the illustrative quotes are presented in Table 8.

**Table 8:** Theme 4: Patient and Medical Environments, and Subthemes with Illustrative Quotes

Themes and Subthemes	Illustrative Quotes
Trust in the physicians	*D12*: “According to what the doctor prescribed to me I thought [I trusted] all the time […] I trust the doctors, I know that all this is for me to be in good health” *(Man, Southeast Europe, F/N permit, 0–5 years in dialysis)* *D13*: “I’m not particularly curious, if you give me that [the medication] it’s for my well-being […], if the doctor told me to do so, I do that […] I always said that the doctors studied for it” *(Man, Swiss, 0–5 years in dialysis)*
Shared decision making	*D14*: “For amlodipine, I negotiated this regimen because before they [the physicians] told me to take every day and I thought it was a bit ridiculous to take in the morning when I was already hypotensive so I discussed with the physician and we found this solution^”^. *(Woman, Western Europe, B/C permit, 0–5 years in dialysis)*
Migration background	*D12*: “The physicians also told me – go elsewhere for your health because you are at risk – that’s why I came here [to Switzerland] […] I was alone here and I did not know a word in French, it was a very difficult situation […] [Now] I am very happy about my health, about what the physicians did, the nurses, all^”^. *(Man, Southeast Europe, F/N permit, 0–5 years in dialysis)* *D9*: “I didn’t know exactly where I was going, how the patients were managed [in Switzerland], but when I was in [my country], I almost lost my life twice […] for me it was this [migration] or nothing, I was willing to take this risk, I did it^”^. *(Man, Western Asia, F/N permit, 0–5 years in dialysis)*

Half of the patients trusted their physicians, because they prescribe medications to improve patients’ health while ensuring patient security; their medical knowledge and education background were praised (eg “I want to believe”). Physicians were clearly the chosen healthcare provider to discuss problems associated with medications.

A few patients took an active role in their health decisions. They described the process of shared decision-making, with an active and essential contribution of both the patient and the physician. In other situations, the patients preferred not to talk to their physician; they took unilateral decisions about their treatment (eg decreasing or increasing the dosage) while informing their physician purposely in a second time. A patient preferred not to express some personal health and medication beliefs because he was afraid of the physician’s reactions and comments. A few patients also expressed dissatisfaction with the lack of time of healthcare professionals.

A few patients who had experienced a migration (provisionally admitted foreign nationals or asylum seekers) spoke spontaneously about this theme. The patients described their difficult health situation in their country of origin. One patient explained that he was encouraged by his doctors to leave his country because he was at risk. Finally, they reported satisfaction with the quality of care in Switzerland.

### Speech Differences According to the Migration Status

Provisionally admitted foreign nationals and asylum seekers often spoke about a feeling of gratitude and even love towards physicians and health professionals. On their side, Swiss patients reported more that they discussed with physicians if they encountered problems with medications. Moreover, Swiss patients seem to have a better knowledge of their treatment; they were very familiar with the names and the indications of the medications. Finally, Swiss patients were also more likely to express their fears of side effects than resident/foreign nationals and provisionally admitted foreign nationals/asylum seekers.

## Discussion

This qualitative study provided the opportunity to explore in depth the medication management, the knowledge and the perceptions of polypharmacy patients in a chronic dialysis unit in Switzerland. For these patients, taking medication had become a habit, treatment forgetfulness was rare and occurred in particular situations. Perceived necessity of treatment was high although treatment knowledge was heterogeneous. Patients spoke about medication drawbacks such as pill burden and side effects. Although they trusted their physicians, they sometimes took unilateral decisions regarding their treatment. Swiss patients seem to be more involved in shared decision-making. They seem to have a better knowledge, and they were more likely to talk about side effects than provisionally admitted foreign nationals/asylum seekers. Provisionally admitted foreign nationals/asylum seekers expressed more gratitude for the healthcare system.

The presence of the interpreters is a strength of the study, as they made it possible to interview allophone patients and collect their perspectives. Allophone patients are often excluded from studies because of the language barrier.

Our findings on treatment management are similar to those found in the literature; most patients associate medication taking with rituals, such as mealtimes, bedtime, or prayer.^43^,^44^ Patients may forget to take their medications, particularly when they are busy, away from home or have disrupted routines.^45^ Healthcare professionals have an important role in training patients in order to anticipate, prevent and solve problems.^46^

Main difficulties quoted by patients about their medications were pill burden and side effects. Pill burden in patients is among the highest of all chronic diseases (eg 11 oral medications per patient on average in our study).^16^,^47–49^ Interestingly, Swiss patients were more likely to talk about side effects compared to resident/settled foreign nationals and provisionally admitted foreign nationals/asylum seekers. Our results suggest that there are various perceptions of side effects, with Swiss patients being more sensitive to this topic and vulnerable to their perceived risk than migrant patients. More research on this topic is needed as literature is scarce, yet such results are important to guide healthcare professionals.

Even though patients in our study perceived their treatment as necessary, some of them ranked medications in order of importance. This result is coherent with other findings; in previous studies, patients also often ranked medications, and their adherence was lower for medications that did not produce visible effects or for medications prescribed for less serious diseases.^21^,^50^,^51^

Several lessons can be learned from our findings, which are consistent with the literature. Firstly, they suggest that it is essential to assess the importance patients attach to each individual treatment they have to take.^51^ Projecting oneself into a virtual situation of nonadherence, as some patients did spontaneously during our interviews (“what would happen if I do not take the medications?”), could be used as a communication and motivational technique to evaluate how patients perceive treatment necessity.^52–55^ To provide tailored education, it is essential to evaluate patients’ health literacy and to use the teach-back method to find the best way to convey health messages.^56–60^ Finally, healthcare professionals have to acquire transcultural clinical skills to improve migrant patients’ quality of care.^61^,^62^ Transcultural clinical skills are a set of attitudes, knowledge and know-how that allow healthcare professionals to provide quality care to patients of different origins.^61^ The acquisition of transcultural clinical skills improves the quality of management of migrant and foreign patients. Moreover, this limits the risk of inequities in terms of access and quality of care.^61^

Despite a relationship of trust, some patients made decisions about their treatment on their own and they only informed their physicians about them in a second time. Such patients seem to need a certain level of autonomy to experiment and report personal experiences. In that respect, motivational interviewing, designed to empower patients to self-manage their care, could help strengthen the dialogue and the mutual trust between patients and healthcare professionals.^52–55^ According to a systematic review, a high level of self-efficacy and internal health control beliefs empower patients to build up optimal medication adherence but, at the same time, patients must also share the control with healthcare providers.^63^ This approach is called “joint empowerment”. More studies are needed to understand how this control is best shared between patients and health professionals (physicians, pharmacists, nurses).^63^

This study has some limitations. Firstly, some patients already knew the interviewer (JDC) as a pharmacist and this could have potentially influenced the content of their interview (eg social desirability bias).^64^ However, JDC clarified her role as a researcher before the inclusion process. Moreover, no difference in the themes were identified between patients that knew or did not know the interviewer. Secondly, the medication adherence components (initiation, implementation and discontinuation) have not been distinguished during the interviews. Thirdly, a single 1-hour interview may not be sufficient to allow participants to unfold their deepest beliefs without restraint. In the future, it would be interesting to repeat the interview at defined intervals to enrich the content. The theme of survival in a foreign country, with all the uncertainties regarding their future, deserves to be more explored.

## Conclusion

To our knowledge, the DIANA study is the first study looking at medication adherence in patients on dialysis in Switzerland that takes into account patients’ migration status.

Medication adherence among dialysis patients depends not only on their understanding and perception of the necessity of treatment but also on their environmental context, including routines and healthcare relationships. As the migration background plays a crucial role in shaping patients’ attitudes toward the healthcare system and their treatment, future interventions should focus on enhancing patient education and should further investigate the concepts of joint empowerment and transcultural clinical skills in the field of medication adherence.

## References

[1] Boerstra BA, Boenink R, Astley ME, et al. The ERA registry annual report 2021: a summary. *Clin Kidney J*. 2024;17(2):sfad281. doi:10.1093/ckj/sfad281 38638342 PMC11024806

[2] Burnier M, Martin PY. Dialyse et réduction des coûts de la santé: des solutions éthiquement acceptables ? *Rev Med Suisse*. 2018;14(595):403–404. doi:10.53738/REVMED.2018.14.595.0403 29465870

[3] Clark S, Farrington K, Chilcot J. Nonadherence in dialysis patients: prevalence, measurement, outcome, and psychological determinants. *Semin Dial*. 2014;27(1):42–49. doi:10.1111/sdi.12159 24164416

[4] Xing Z, Wang Y, Li H, et al. Theory-based interventions to promote fluid intake adherence among dialysis patients: a systematic review. *Res Theory Nurs Pract*. 2019;33(4):357–391. doi:10.1891/1541-6577.33.4.357 31666394

[5] Kim Y, Evangelista LS. Relationship between illness perceptions, treatment adherence, and clinical outcomes in patients on maintenance hemodialysis. *Nephrol Nurs J*. 2010;37(3):271–280. 20629465 PMC3172671

[6] Beerendrakumar N, Ramamoorthy L, Haridasan S. Dietary and fluid regime adherence in chronic kidney disease patients. *J Caring Sci*. 2018;7(1):17–20. doi:10.15171/jcs.2018.003 29637052 PMC5889793

[7] Nadri A, Khanoussi A, Hssaine Y, Chettati M, Fadili W, Laouad I. Effect of a hemodialysis patient education on fluid control and dietary. *Nephrol Ther*. 2020;16(6):353–358. doi:10.1016/j.nephro.2020.03.011 33132076

[8] Murali KM, Mullan J, Roodenrys S, Hassan HC, Lambert K, Lonergan M. Strategies to improve dietary, fluid, dialysis or medication adherence in patients with end stage kidney disease on dialysis: a systematic review and meta-analysis of randomized intervention trials. *PLoS One*. 2019;14(1):e0211479. doi:10.1371/journal.pone.0211479 30695068 PMC6350978

[9] Vr V, Kaur Kang H. The worldwide prevalence of nonadherence to diet and fluid restrictions among hemodialysis patients: a systematic review and meta-analysis. *J Ren Nutr*. 2022;32(6):658–669. doi:10.1053/j.jrn.2021.11.007 34923113

[10] Vrijens B, De Geest S, Hughes DA, et al. A new taxonomy for describing and defining adherence to medications. *Br J Clin Pharmacol*. 2012;73(5):691–705. doi:10.1111/j.1365-2125.2012.04167.x 22486599 PMC3403197

[11] Ghimire S, Castelino RL, Lioufas NM, Peterson GM, Zaidi ST. Nonadherence to medication therapy in haemodialysis patients: a systematic review. *PLoS One*. 2015;10(12):e0144119. doi:10.1371/journal.pone.0144119 26636968 PMC4670103

[12] Aspden T, Wolley MJ, Ma TM, et al. Understanding barriers to optimal medication management for those requiring long-term dialysis: rationale and design for an observational study, and a quantitative description of study variables and data. *BMC Nephrol*. 2015;16:102. doi:10.1186/s12882-015-0097-2 26162369 PMC4499205

[13] Griva K, Lai AY, Lim HA, Yu Z, Foo MW, Newman SP. Non-adherence in patients on peritoneal dialysis: a systematic review. *PLoS One*. 2014;9(2):e89001. doi:10.1371/journal.pone.0089001 24586478 PMC3934877

[14] Tesfaye WH, Erku D, Mekonnen A, et al. Medication non-adherence in chronic kidney disease: a mixed-methods review and synthesis using the theoretical domains framework and the behavioural change wheel. *J Nephrol*. 2021;34(4):1091–1125. doi:10.1007/s40620-020-00895-x 33559850

[15] Mechta Nielsen T, Marott T, Hornum M, Feldt-Rasmussen B, Kallemose T, Thomsen T. Non-adherence, medication beliefs and symptom burden among patients receiving hemodialysis -a cross-sectional study. *BMC Nephrol*. 2023;24(1):321. doi:10.1186/s12882-023-03371-3 37891566 PMC10604404

[16] Wileman V, Farrington K, Wellsted D, Almond M, Davenport A, Chilcot J. Medication beliefs are associated with phosphate binder non-adherence in hyperphosphatemic haemodialysis patients. *Br j health psychol*. 2015;20(3):563–578. doi:10.1111/bjhp.12116 25209368

[17] Chater AM, Parham R, Riley S, Hutchison AJ, Horne R. Profiling patient attitudes to phosphate binding medication: a route to personalising treatment and adherence support. *Psychol Health*. 2014;29(12):1407–1420. doi:10.1080/08870446.2014.942663 25012529

[18] Yu ZL, Lee VY, Kang AW, et al. Rates of intentional and unintentional nonadherence to peritoneal dialysis regimes and associated factors. *PLoS One*. 2016;11(2):e0149784. doi:10.1371/journal.pone.0149784 26919323 PMC4769138

[19] Griva K, Ng HJ, Loei J, Mooppil N, McBain H, Newman SP. Managing treatment for end-stage renal disease--a qualitative study exploring cultural perspectives on facilitators and barriers to treatment adherence. *Psychol Health*. 2013;28(1):13–29. doi:10.1080/08870446.2012.703670 22780853

[20] Lam LW, Lee DT, Shiu AT. The dynamic process of adherence to a renal therapeutic regimen: perspectives of patients undergoing continuous ambulatory peritoneal dialysis. *Int J Nurs Studies*. 2014;51(6):908–916. doi:10.1016/j.ijnurstu.2013.10.012 24210362

[21] McKillop G, Joy J. Patients’ experience and perceptions of polypharmacy in chronic kidney disease and its impact on adherent behaviour. *J Renal Care*. 2013;39(4):200–207. doi:10.1111/j.1755-6686.2013.12037.x 24245971

[22] Rifkin DE, Laws MB, Rao M, Balakrishnan VS, Sarnak MJ, Wilson IB. Medication adherence behavior and priorities among older adults with CKD: a semistructured interview study. *Am J Kidney Dis*. 2010;56(3):439–446. doi:10.1053/j.ajkd.2010.04.021 20674113 PMC2935303

[23] Ghimire S, Castelino RL, Jose MD, Zaidi STR. Medication adherence perspectives in haemodialysis patients: a qualitative study. *BMC Nephrol*. 2017;18(1):167. doi:10.1186/s12882-017-0583-9 28532480 PMC5440949

[24] Tijerina MS. Psychosocial factors influencing Mexican-American women’s adherence with hemodialysis treatment. *Soc Work Health Care*. 2006;43(1):57–74. doi:10.1300/J010v43n01_04 16723335

[25] Rizzolo K, Gonzalez Jauregui R, Barrientos I, et al. Barriers and facilitators to home dialysis among latinx patients with kidney disease. *JAMA Netw Open*. 2023;6(8):e2328944. doi:10.1001/jamanetworkopen.2023.28944 37581885 PMC10427944

[26] Martin Y, Collet TH, Bodenmann P, et al. The lower quality of preventive care among forced migrants in a country with universal healthcare coverage. *Preventive Med*. 2014;59:19–24. doi:10.1016/j.ypmed.2013.11.006 24262974

[27] Jaboyedoff M, Genton B, Masserey E, Bodenmann P, Rimaz R, de Valliere S. Hepatitis B and migrants: should we do better? *Rev Med Suisse*. 2014;10(421):617–621. doi:10.53738/REVMED.2014.10.421.0617 24701715

[28] Bodenmann P, Bossart R, Di Bernardo N, et al. Managing diversity in Swiss Health care. *Rev Med Suisse*. 2014;10(451):2222–2225. doi:10.53738/REVMED.2014.10.451.2222 25603570

[29] Jaton L, Kritikos A, Bodenmann P, Greub G, Merz L. European migrant crisis and reemergence of infections in Switzerland. *Revue med suisse*. 2016;12(514):749–753. 27263151

[30] Blaser J, Ambresin AE, Monnat M, et al. Assessing the plight of young unaccompanied refugees. *Swiss Med Weekly*. 2017:147:w14547. doi:10.4414/smw.2017.14547. 29185239

[31] Kristensen E, Rausa F, Heiniger M. *Rapport statistique sur l’intégration de la population issue de la migration*. 2017. *Statistique de la Suisse*. Available from: https://www.bfs.admin.ch/bfs/fr/home/statistiques/population/migration-integration/indicateurs-integration.assetdetail.2546311.html. Accessed June 30, 2025.

[32] Bodenmann P, Jackson Y, Wolff H. *Vulnérabilités, équité et santé*. RMS éditions / Médecine et Hygiène; 2018.

[33] Nguyen DQ. Qui sont ces 25% d’étrangers en Suisse. Available from: https://www.swissinfo.ch/fre/s%C3%A9rie-migration-partie-1-_deux-millions-d-%C3%A9trangers-en-suisse-mais-qui-sont-ils/42409190. Accessed June 30, 2025.

[34] SEM Sécrétariat d’État aux migrations SEM. *Statistiques annuelles sur l’immigration 2022*. 2023. Available from: https://www.google.com/url?sa=t&rct=j&q=&esrc=s&source=web&cd=&ved=2ahUKEwinxoOL0LyMAxV3hv0HHWg4Jk0QFnoECCIQAQ&url=https%3A%2F%2Fwww.sem.admin.ch%2Fdam%2Fsem%2Ffr%2Fdata%2Fpubliservice%2Fstatistik%2Fauslaenderstatistik%2Fmonitor%2F2022%2Fstatistik-zuwanderung-2022-jahr.pdf.download.pdf%2Fstatistik-zuwanderung-2022-jahr-f.pdf&usg=AOvVaw3sLCT817MFYBmVmMiJ7EFz&opi=89978449. Accessed June 30, 2025.

[35] SEM Sécrétariat d’État aux migrations SEM. Asylum Statistics. 2025. Available from: https://www.sem.admin.ch/sem/fr/home/publiservice/statistik/asylstatistik/archiv/2025/02.html. Accessed June 30, 2025.

[36] Dotta-Celio J, Ballabeni P, Maeder S, et al. In press: migration status and medication adherence in chronic dialysis patients: a cross-sectional study in Switzerland.

[37] Tong A, Sainsbury P, Craig J. Consolidated criteria for reporting qualitative research (COREQ): a 32-item checklist for interviews and focus groups. *Int J Qual Health Care*. 2007;19(6):349–357. doi:10.1093/intqhc/mzm042 17872937

[38] De Geest S, Zullig LL, Dunbar-Jacob J, et al. ESPACOMP medication adherence reporting guideline (EMERGE). *Ann Internal Med*. 2018;169(1):30–35. doi:10.7326/m18-0543 29946690 PMC7643841

[39] Fisher JD, Fisher WA, Amico KR, Harman JJ. An information-motivation-behavioral skills model of adherence to antiretroviral therapy. *Health Psychol*. 2006;25(4):462–473. doi:10.1037/0278-6133.25.4.462 16846321

[40] Munro SA, Lewin SA, Swart T, Volmink J. A review of health behaviour theories: how useful are these for developing interventions to promote long-term medication adherence for TB and HIV/AIDS? 10.1186/1471-2458-7-104. *BMCPublic Health*. 2007;7(1):104. 10.1186/1471-2458-7-104 PMC1925084 17561997

[41] Martin LR, Haskard-Zolnierek KB, DiMatteo MR. Health behavior change and treatment adherence: evidence-based guidelines for improving healthcare. *Oxford Univ Press*. 2010;212.

[42] Mays N, Pope C. Rigour and qualitative research. Review. *BMJ*. 1995;311(6997):109–112. doi:10.1136/bmj.311.6997.109 7613363 PMC2550154

[43] Battistella M, Fleites R, Wong R, Jassal SV. Development, validation, and implementation of a medication adherence survey to seek a better understanding of the hemodialysis patient. *Clin nephrol*. 2016;85(1):12–22. doi:10.5414/cn108654 26636327

[44] Badawy SM, Shah R, Beg U, Strength HMBH. Medication adherence, and habit-based mobile health interventions across chronic medical conditions: systematic review. *J Med Internet Res*. 2020;22(4):e17883. doi:10.2196/17883 32343250 PMC7218590

[45] Browne T, Merighi JR. Barriers to adult hemodialysis patients’ self-management of oral medications. *Am J Kidney Dis*. 2010;56(3):547–557. doi:10.1053/j.ajkd.2010.03.002 20430501

[46] Hafez G, Aarnio E, Mucherino S, et al. Barriers and unmet educational needs regarding implementation of medication adherence management across Europe: insights from COST action ENABLE. *J Gen Intern Med*. 2024;39(15):2917–2926. doi:10.1007/s11606-024-08851-2 38941058 PMC11576669

[47] Sales I, Bawazeer G, Tarakji AR, et al. Assessment of dietary folate intake and pill burden among Saudi patients on maintenance hemodialysis. *Int J Environ Res Public Health*. 18(23). doi:10.3390/ijerph182312710 PMC8657290 34886434

[48] Paik JM, Zhuo M, York C, Tsacogianis T, Kim SC, Desai RJ. medication burden and prescribing patterns in patients on hemodialysis in the USA, 2013-2017. *Am J Nephrol*. 2021;52(12):919–928. doi:10.1159/000520028 34814147 PMC8979193

[49] Liu X, Chen P, Liu Y, Jia X, Xu D. Medication burden in patients with dialysis-dependent CKD: a systematic review. *Ren Fail*. 2024;46(1):2353341. doi:10.1080/0886022x.2024.2353341 38832502 PMC11151796

[50] Foley L, Larkin J, Lombard-Vance R, et al. Prevalence and predictors of medication non-adherence among people living with multimorbidity: a systematic review and meta-analysis. *BMJ Open*. 2021;11(9):e044987. doi:10.1136/bmjopen-2020-044987 PMC8413882 34475141

[51] Reach G, Calvez A, Sritharan N, et al. Patients’ perceived importance of medication and adherence in polypharmacy, a quantitative, cross-sectional study using a questionnaire administered in three doctors’ private practices in France. *Drugs Real World Outcomes*. 2023;10(2):309–320. doi:10.1007/s40801-023-00361-7 36997772 PMC10232699

[52] Rollnick S, Miller WR. What is the motivational interviewing? In: *Behavioural and Cognitive Psychotherapy*. Vol. 23. NOT IN FILE; 1995:325–334. 10.1017/S1352465809005128 19364414

[53] Frost H, Campbell P, Maxwell M, et al. Effectiveness of Motivational Interviewing on adult behaviour change in health and social care settings: a systematic review of reviews. *PLoS One*. 2018;13(10):e0204890. doi:10.1371/journal.pone.0204890 30335780 PMC6193639

[54] Bischof G, Bischof A, Rumpf HJ. Motivational interviewing: an evidence-based approach for use in medical practice. *Dtsch Arztebl Int*. 2021;118(7):109–115. doi:10.3238/arztebl.m2021.0014 33835006 PMC8200683

[55] Palacio A, Garay D, Langer B, Taylor J, Wood BA, Tamariz L. motivational interviewing improves medication adherence: a systematic review and meta-analysis. *J Gen Intern Med*. 2016;31(8):929–940. doi:10.1007/s11606-016-3685-3 27160414 PMC4945560

[56] Baker DW. The meaning and the measure of health literacy. *J Gen Intern Med*. 2006;21(8):878–883. doi:10.1111/j.1525-1497.2006.00540.x 16881951 PMC1831571

[57] Katz SJ, Hawley S. The value of sharing treatment decision making with patients: expecting too much? *JAMA*. 310(15):1559–1560. doi:10.1001/jama.2013.278944 24061082

[58] Boonstra MD, Reijneveld SA, Foitzik EM, Westerhuis R, Navis G, de Winter AF. How to tackle health literacy problems in chronic kidney disease patients? A systematic review to identify promising intervention targets and strategies. *Nephrol Dial Transp*. 2021;36(7):1207–1221. doi:10.1093/ndt/gfaa273 PMC8237988 33351936

[59] Brown C, Dotson B, Montgomery J, Sutterfield C, Maharaj G. Evaluating the effectiveness of using the teach-back method to improve the health literacy of individuals in the community. *J Community Health Nurs*. 1–8. doi:10.1080/07370016.2024.2399347 39252389

[60] Yen PH, Leasure AR. Use and effectiveness of the teach-back method in patient education and health outcomes. *Fed Pract*. 2019;36(6):284–289. 31258322 PMC6590951

[61] Althaus F, Hudelson P, Domenig D, Green AR, Bodenmann P. Compétences cliniques transculturelles et pratique médicale. Quels besoins, quels outils, quel impact?. *Forum Med Suisse*. 2010;10:79–83. doi:10.4414/fms.2010.07077

[62] Antón-Solanas I, Tambo-Lizalde E, Hamam-Alcober N, et al. Nursing students’ experience of learning cultural competence. *PLoS One*. 2021;16(12):e0259802. doi:10.1371/journal.pone.0259802 34919540 PMC8683022

[63] Nafradi L, Nakamoto K, Schulz PJ. Is patient empowerment the key to promote adherence? A systematic review of the relationship between self-efficacy, health locus of control and medication adherence. *PLoS One*. 2017;12(10):e0186458. doi:10.1371/journal.pone.0186458 29040335 PMC5645121

[64] Bispo Júnior JP. Social desirability bias in qualitative health research. *Rev Saude Publica*. 2022;56:101. doi:10.11606/s1518-8787.2022056004164 36515303 PMC9749714

[65] Celio J. *Études en adhésion thérapeutique: Avancements dans la collaboration interprofessionnelle et dans les domaines cliniques de l’anticoagulation, de l’insuffisance rénale chronique et du diabète*. Geneva University; 2019. https://archive-ouverte.unige.ch/unige:120060.

